# Computerized Assessment of the Tumor-stromal Ratio and Proposal of a Novel Nomogram for Predicting Survival in Invasive Breast Cancer

**DOI:** 10.7150/jca.55750

**Published:** 2021-04-19

**Authors:** Xu Qian, Feng Xiao, Yuan-Yuan Chen, Jing-Ping Yuan, Xiao-Hong Liu, Lin-Wei Wang, Bin Xiong

**Affiliations:** 1 Hubei Key Laboratory of Tumor Biological Behaviors, Hubei Cancer Clinical Study Center, Wuhan, China, 430071.; 2 Department of Gastrointestinal Surgery, Zhongnan Hospital of Wuhan University, Wuhan, China, 430071.; 3 Department of Radiology, Zhongnan Hospital of Wuhan University, Wuhan, China, 430071.; 4 Department of Radiation and Medical Oncology, Zhongnan Hospital of Wuhan University, Wuhan, China, 430071.; 5 Department of Pathology, Renmin Hospital of Wuhan University, 430060 Wuhan, China.

**Keywords:** Breast cancer, Tumor stromal ratio, Computerized assessment, Nomogram.

## Abstract

**Background:** Various studies have verified the prognostic significance of the tumor-stromal ratio (TSR) in several types of carcinomas using manually assessed H&E stained histologic sections. This study aimed to establish a computerized method to assess the TSR in invasive breast cancer (BC) using immunohistochemistry (IHC)-stained tissue microarrays (TMAs), and integrate the TSR into a novel nomogram for predicting survival.

**Methods:** IHC-staining of cytokeratin (CK) was performed in 7 prepared TMAs containing 240 patients with 480 invasive BC specimens. The ratio of tumor areas and stromal areas was determined by the computerized method, and categorized as stroma-low and stroma-high groups using the X-tile software. The prognostic value of the TSR at 5-year disease free survival (5-DFS) in each subgroup was analyzed. Univariate and multivariate analyses were performed and a novel nomogram for predicting survival in invasive breast cancer was established and assessed.

**Results:** The newly developed computerized method could accurately recognize CK-labeled tumor areas and non-labeled stromal areas, and automatically calculate the TSR. Stroma-low and stroma-high accounted for 38.8% (n = 93) and 61.2% (n = 147) of the cases, according to the cut-off value of 55.5% for stroma ratio. The Kaplan-Meier analysis showed that patients in the stroma-high group had a worse 5-DFS compared to patients in the stroma-low group (*P* = 0.031). Multivariable analysis indicated that the T stage, N status, histological grade, ER status, HER-2 gene, and the TSR were potential risk factors of invasive BC patients, which were included into the nomogram (*P* < 0.10 for all). The nomogram was well calibrated to predict the probability of 5-DFS and the C-index was 0.817, which was higher than any single predictor. A dynamic nomogram was built for convenient use. The area under the curve (AUC) of the nomogram was 0.870, while that of the TNM staging system was 0.723. The Kaplan-Meier analysis showed that the nomogram had a better risk stratification for invasive BC patients than the TNM staging system.

**Conclusions:** Based on IHC staining of CK on TMAs, this study successfully developed a computerized method for TSR assessment and established a novel nomogram for predicting survival in invasive BC patients.

## Introduction

Breast cancer represents a serious health threat among females worldwide, with an estimated 1.6 million new cases and 520,000 deaths every year according to the GLOBOCAN database 1-2. Although considerable improvements have been achieved over the past decades due to the progress in screening programmes and comprehensive treatments, the disease prevalence and mortality rates of BC remained high over the past few decades 3-4. More prognostic indicators are urgently needed to optimize the risk stratification and contemplate treatment options in invasive BC patients.

Recently, various studies demonstrate that the cancer progression is not only related to biological behavior of tumor cells but also to tumor microenvironment, which includes surrounding blood vessels, the extracellular matrix, other normal cells, and signaling molecules 5-6. Tumor stroma, as an important component of tumor microenvironment, plays a pivotal role during tumor initiation, progression, and metastasis. Non-malignant cells in the tumor stoma can promote growth and survival of malignant cells through secreting various growth factors, chemokines, and cytokines 7-8. Therefore, the tumor-stromal ratio, a parameter representing the proportion of tumor-associated stroma, was introduced to the field of cancer research 9-10.

A reliable method for assessment is the basis to explore prognosis of the TSR. In our previous study, CK, an epithelial-specific marker, was applied to specifically label the tumor cells. Compared with H&E staining, IHC staining of CK resulted in a clear color contrast of brown tumor areas and off-white stromal areas, which made it easier to assess the TSR through visual scoring, and the TSR was proved to be of prognostic value for invasive BC. However, with Cohen's kappa value of 0.77, the manual method was still criticized due to its intra- and inter-observer variations 11. To overcome this disadvantage, we established a new computerized method for tumor/stroma recognition and TSR assessment. This method could recognize CK-labeled tumor areas and non-labeled stromal areas, and automatically calculate the TSR.

As a statistical predictive model, a nomogram estimates individualized risk on the basis of clinic-pathologic factors. It assigns relative risk score to each predictor according to its contribution for the prognosis. Owing to its advantage to predict the incidence rate or survival rate through a scoring system rather than calculating a complex formula, the nomogram has emerged as a simpler, yet more advanced method over traditional staging systems 12-13.

Given this, this study developed a computerized method to assess the TSR in invasive BC using CK stained TMAs. Furthermore, a novel nomogram containing the TSR for predicting survival in invasive BC was established and assessed.

## Materials and methods

### Patients and specimens

The clinical database of BC of our center has been the data source of several clinical and translational studies 14-15. From the database, 240 invasive BC specimens were collected and TMAs were constructed according to the same criteria. Major clinic-pathologic characteristics were available, including the tumor stage, location, histological type, lymph node status, ER, PR and HER2 status. TNM staging and histological grading were determined according to the 8th edition of the UICC/AJCC TNM classification 16 and the WHO histological grading 17. The failure event of the follow-up study was locoregional recurrence or metastasis. 5-year disease-free survival (DFS) was collected from the case file of each patient. Approval of the study protocol was granted by the Institutional Ethics Committee of Zhongnan Hospital of Wuhan University (Scientific Ethical Approval NO.2017057). The study was undertaken according to the ethical standards of the World Medical Association Declaration of Helsinki.

### Tissue microarrays construction

TMAs were constructed using standard procedures in collaboration with Shanghai Outdo Biotech Co. Ltd. (Shanghai, China), as previously described 18. For all specimens, diagnostic 4μm H&E-stained sections were obtained and inspected, and the most invasive tumor areas containing both tumor cells and tumor stroma were identified. Corresponding areas were marked on the original FFPE block for cutting. Two cores were taken from marked areas of each paraffin block using punch cores. The cores were then deposited into recipient paraffin blocks with 70 cylinders. Seven TMAs blocks containing 480 cores were constructed and cut into 4μm sections, with one slide every 50 retained for H&E staining and quality control.

### IHC staining of CK

IHC staining of CK was performed in our previous study 11. Firstly, TMA slides were deparaffinized and rehydrated with successive washes in dimethylbenzene (15min) and alcohol ((100, 90, and 70%, 5min each). Epitope retrieval was performed using 0.01 mol/L citrate buffer (pH 6.0) heated by a microwave oven (95°C, 15min). The endogenous peroxidase activity was blocked using 3% H2O2 (10min) at room temperature. The blocking antibody (2% BSA) was applied to decrease background intensity. All slides were incubated with the anti-pan CK antibody (mouse anti-human, ZSGB-BIO, China, clone AE1/AE3; 1:100 dilution) overnight at 4℃, and then with corresponding secondary antibody (1:250 dilution) for 30 min at 37 °C. Diaminobenzidine was added as the chromogen and counterstaining was performed with hematoxylin for 2 min. Following dehydration, the slides were sealed with resin mount.

### Image acquisition and assessment of TSR

TMAs were scanned with the Aperio VERSA automated slide scanner (Leica Biosystems Imaging, Buffalo Grove, IL). Digital images of cores in TMAs were obtained using the Aperio Image Scope Software (Leica Biosystems Imaging, Buffalo Grove, IL). Using a computerized TSR assessment approach, digital images of cores in TMAs were assessed to calculate the proportion of stroma areas. The computerized approach comprises the following steps, ①Transforming the color image into grayscale image; ②Calculating the image gradient using edge and sobel operators to detect the contours of objects; ③Obtaining the tumor objects in the image using morphological operation (dilate-> fill-> erode) with small objects eliminated; ④Staining the tumor objects and non-tumor areas in the image with different colors (colored image); ⑤Increasing the image contrast differences using histogram equalization for subsequent segmentation; ⑥Performing image segmentation using otsu algorithm; ⑦Obtaining the whole core object in the image (mask map); ⑧Merging the colored image and mask map to get the final image and calculating the TSR, TSR=area of stroma objects (in cyan)/area of the whole core object (tumor objects (in magenta) + stromal objects (in cyan)). The field of highest stromal percentage from two cores of each specimen were considered crucial.

### Statistical analysis

The cut-off point of TSR was determined using the X-tile software based on the best *P* value principle. Distribution of the clinic-pathologic factors between stroma-low and stroma-high groups was evaluated using Pearson χ2 test or Fisher's exact test. The Kaplan-Meier method was performed to analyze the 5-DFS. The log-rank test was applied for comparison between the curves. Unadjusted HRs (hazard ratios) and 95% CIs of TSR for 5-DFS in each subgroup were calculated using by Cox proportional hazard analysis. The Cox regression model was used to perform univariate and multivariate analyses for 5-DFS. In the univariate analysis, potential risk factors were selected. In the multivariate analysis, three selection procedures (enter, forward, backward) were performed to select variables into the best-fit model. A statistical significance level of 0.10 was used, which can reduce the impact caused by the small sample size. The above statistical analyses were performed using IBM SPSS statistics (version 23.0 for Windows).

Based on the results of multivariate regression and clinical experience, a baseline nomogram was constructed to generate probability of 5-DFS. The performance of the nomogram included its discrimination and calibration. Discrimination was defined as a model's ability to correctly distinguish non-events and events, which was quantified using a concordance index (C-index). Calibration measures how closely the predicted survival rate agree with the actual survival rate, which was evaluated by the calibration plot. In addition, the receiver operating characteristic (ROC) curve was used to compare the discrimination ability of the nomogram with the TNM staging system. The Kaplan-Meier curve and the Log-rank test was performed to estimate the probability of 5-DFS between risk subgroups. The above statistical analyses were performed using R 3.6.3 software (https://cran.r-project.org/). The dynamic nomogram was built through package “DynNom”.

## Results

### IHC staining images and computerized assessment results

Typical IHC staining images and corresponding computerized assessment results are shown in Fig. 2. IHC staining of CK makes a strong color contrast of brown tumor areas and off-white stromal areas (A1 and B1). After computerized recognition, tumor areas were marked in magenta, stromal areas in cyan, and none-cell areas in black (A2 and B2). Panels A1, A2 are examples of high stroma, with an estimated TSR of 78.1%. Panels B1, B2 are examples of low stroma, with an estimated TSR of 37.9%.

### Evaluation of tumor-stromal ratio

As the TSR quantified by the computerized method was a continuous variable, the X-tile software based on the best *P* value principle was adopted to identify the optimal cut-off point of TSR. A cut-off point of 55.5% was used to categorize patients into stroma-low (TSR ≤ 55.5%) and stroma-high (TSR > 55.5%) groups. Among 240 specimens, 38.8% were determined as stroma-low and 61.2% as stroma-high.

### Correlation between TSR and major clinic-characteristics

There were 240 invasive BC patients included in the study. The median age was 48 years (range, 29-78 years) at the date of surgery. **Table 1** listed the major clinic-pathological characteristics stratified for stroma-low and stroma-high groups. The TSR was significantly associated with menopausal status (*P* = 0.031), but not with age (*P* = 0.244), histological type (*P* = 0.514), T stage (*P* = 0.629), N status (*P* = 0.205), histological grade (*P* = 0.622), ER status (*P* = 0.277), PR status (*P* = 0.499), and HER2 gene status (*P* = 0.090) (**Table 1**).

### Prognosis of BC patients according to TSR

For the entire patient cohort (n = 240), the 5-year disease free survival rate was 62.0%. As expected, those conventional factors were associated with invasive BC patients' 5-DFS (*P* < 0.05 for all) (**Supplementary Table 1**), including T stage, N status, histological grade, histological type, ER, PR, HER-2 status, and menopausal status. The survival curve of patients with high or low TSR are shown in **Fig. 3**. A worse DFS was found for patients with high stroma as compared to patients with low stroma (*χ2*=4.659, *P* = 0.031), with 5-year disease free survival rate of, respectively, 56.5 vs. 71.0%. The result indicated that the TSR might be a prognostic predictor for invasive BC, and patients with stroma-rich tumors showed a trend toward a worse outcome.

### Subgroup analysis of the TSR associated with 5-DFS

Prognostic significance of the TSR for 5-DFS was analyzed in each subgroup (**Fig. 4**). For the whole cohort, a better 5-DFS was found for patients with low stroma as compared to patients with high stroma (HR 0.61; 95% CI 0.38-0.99; *P* = 0.034). Subgroup analysis demonstrated that the TSR was significantly associated with 5-DFS in invasive ductal carcinoma (HR 0.61; 95% CI 0.38-0.99; *P* = 0.045), N positive (HR 0.57; 95% CI 0.34-0.97; *P* = 0.037), ER negative (HR 0.43; 95% CI 0.25-0.74; *P* = 0.002) and HER2 gene non-amplification groups (HR 0.51; 95% CI 0.28-0.90; *P* = 0.021). In addition, a non-significant correlation between low stroma and better 5-DFS was observed (*P* > 0.05) in groups of age, menopausal status, histological grade, and PR status. Notably, HR of the T1 stage and histological grade I groups had a very broad the confidence interval, probably caused by the relatively small sample size or wide sample variability.

### Univariable and multivariable analysis of the TSR and other parameters

The relationship between all clinic-characteristics and 5-DFS was investigated in univariate analyses. Factors that were included into the multivariate model predicting 5-DFS were T stage (*P* < 0.001), N status (*P* < 0.001), histological grade (*P* < 0.001), ER status (*P* < 0.001), PR status (*P* = 0.006), HER2 gene (*P* < 0.001), and the TSR (*P* = 0.034). Factors that remained in the multivariate model for the construction of nomogram were T stage, N status, histological grade, ER status, HER2 gene, and the TSR (*P* < 0.10 for all) (**Table 2**).

### Construction and validation of the prognostic nomogram

Based on the results of multivariate regression and clinical experience, a nomogram was constructed to predict the probability of disease-free survival at the follow-up of 5 years for invasive BC patients (**Fig. 5**). According to the contribution of each predictor for the prognosis (scaled by the proportional regression coefficient of each predictor), relative risk score was assigned to each predictor 12. When using the nomogram, an upward vertical line was drawn from the covariate to the points bar to calculate points. The detailed points of each variable were as follows, T stage (T1: 0.0, T2: 65.1, T3: 87.0), N status (negative: 0.0. positive: 93.4), histological grade (I: 0.0, II: 27.3, III: 100.0), ER status (positive: 0.0, negative: 36.34), HER-2 gene (negative: 0.0, positive: 49.6), and the TSR (stroma-low: 0.0, stroma-high: 30.8). Based on the sum of the covariate points, a downward vertical line was drawn to confirm the probability of 5-DFS. Furthermore, the dynamic version of this nomogram (**Fig. S1**) was established, which could assist users in obtaining probability of disease-free survival at any time within 60 months. The C-index of the nomogram was 0.817 (95% CI: 0.775-0.858), which indicated a good discriminative ability. The calibration plot for predicting 5-year DFS probability also showed favorable consistency between nomogram predictions and observed outcomes (**Fig. 6A**). In addition, we compared the predictive ability of the nomogram with included independent prognostic factors. The C-indices of T stage, N status, histological grade, ER status, HER2 gene and TSR were significantly lower than the C-index of the nomogram (0.817, 95% CI: 0.775-0.858) (**Table 2**).

### Comparison of in the predictive value of the nomogram and TNM staging system

The nomogram had a better ability to predict recurrence as compared to TNM staging system (*P* < 0.001), with an AUC of, respectively, 0.870 (95% CI: 0.823-0.917) vs. 0.723 (95% CI: 0.657-0.790) (**Fig. 6B**). Meanwhile, the sum of the covariate points was calculated for each patient. Through the X-tile software, optimum cut-off points of the total points for three groups were identified (222.2, 316.8). Patients with total points ≤ 222.2, 222.2 < total points ≤ 316.8, and total points > 316.8 were categorized as I (n=144), II (n=64), and III (n=32) groups, respectively. Kaplan-Meier curves showed that the nomogram had a better ability to distinguish BC patients into three groups with different prognoses (χ2=128.361, *P* < 0.001 for I/II and II/III) than the TNM staging system (χ2=59.657, *P* = 0.015 for I/II, *P* < 0.001 for II/III) (**Fig. 7**).

## Discussion

The tumor stroma consists of multiple components, which play a vital role in the tumor progression. TSR, as a new parameter which represents the amount of tumor-associated stroma, has been proved to be significant in prognosis evaluation of different cancer types 21-23. Our data indicated that the TSR might be a prognostic predictor for invasive BC, and patients with stroma-rich tumors showed a trend toward a worse outcome.

Accurate assessment of the TSR is the key to appreciating its prognostic value. Currently, the TSR is largely assessed in the hematoxylin-eosin (HE) staining section with two methods, visual eyeballing 21-22 and point counting 23-24. Visual eyeballing is a manual method with two-steps to determine the TSR. Firstly, at low magnification, the most invasive tumor areas were selected. Then, at high magnification, image fields containing both stroma and tumor are assessed and TSR is scored per tenfold percentage. The other is a semi-automated point counting method. A sample of 300 random points are superimposed on the selected area. Under each of the 300 points, the histopathology is categorized as 'tumor,' 'stroma,' or 'unclassified (necrosis, blood vessels, inflammation, blank areas).' The TSR is expressed as the proportion of 'stroma' under the 300 points, compared with all points per section.

**Figure 1:**
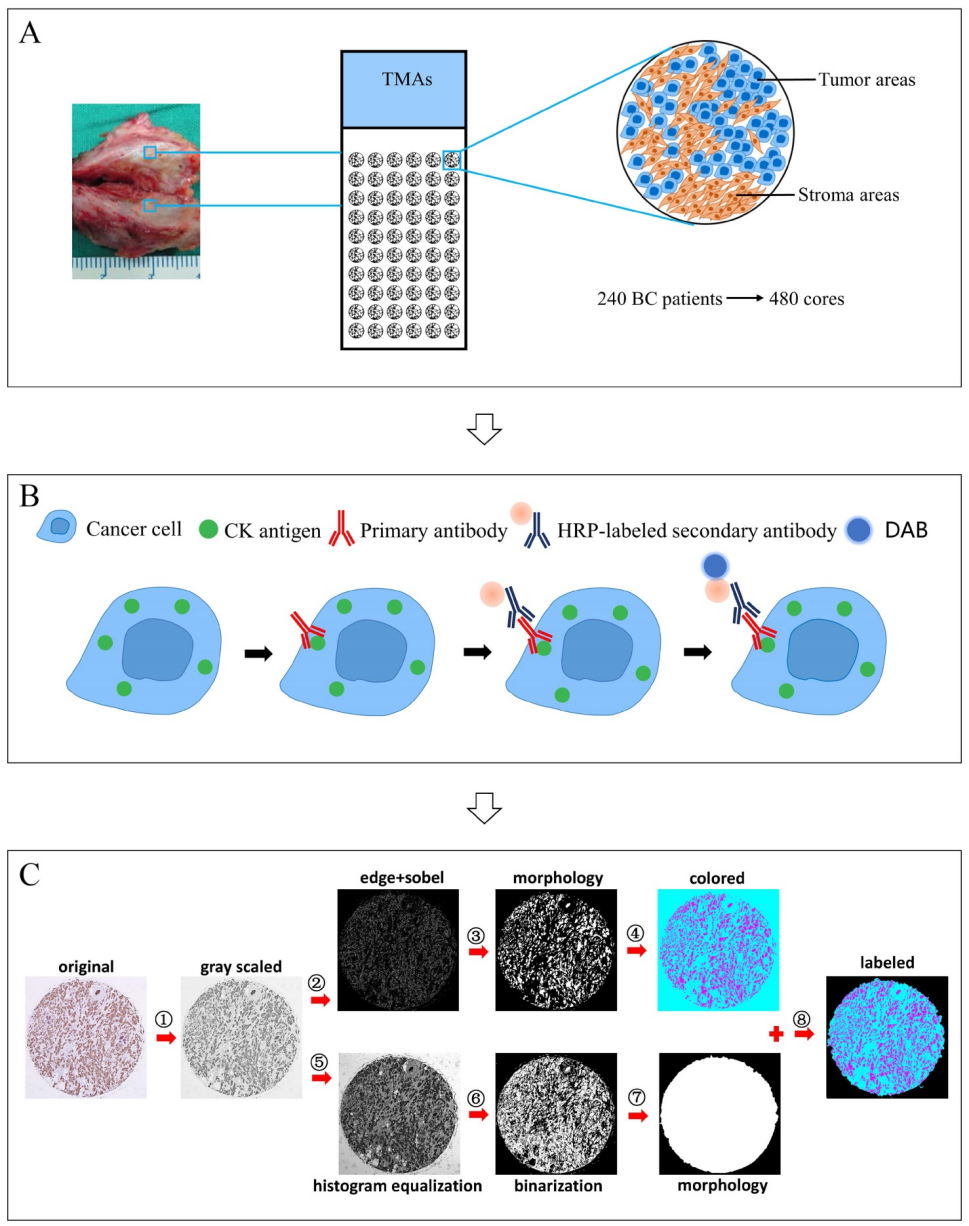
Major technical procedures of this study. **Panel A**, TMAs with 480 cores were constructed using 240 cases of breast cancer specimens. **Panel B**, IHC staining of CK was performed. **Panel C**, computerized TSR assessment was performed, involving the following steps, ①Transforming the color image into grayscale image; ②Calculating the image gradient using edge and sobel operators to detect the contours of objects; ③Obtaining the tumor objects in the image using morphological operation (dilate-> fill-> erode) with small objects eliminated; ④Staining the tumor objects and non-tumor areas in the image with different colors (colored image); ⑤Increasing the image contrast differences using histogram equalization for subsequent segmentation; ⑥Performing image segmentation using otsu algorithm; ⑦Obtaining the whole core object in the image (mask map); ⑧Merging the colored image and mask map to get the final image and calculating the TSR, TSR=area of stroma objects (in cyan)/area of the whole core object (tumor objects (in magenta) + stromal objects (in cyan)).

**Figure 2:**
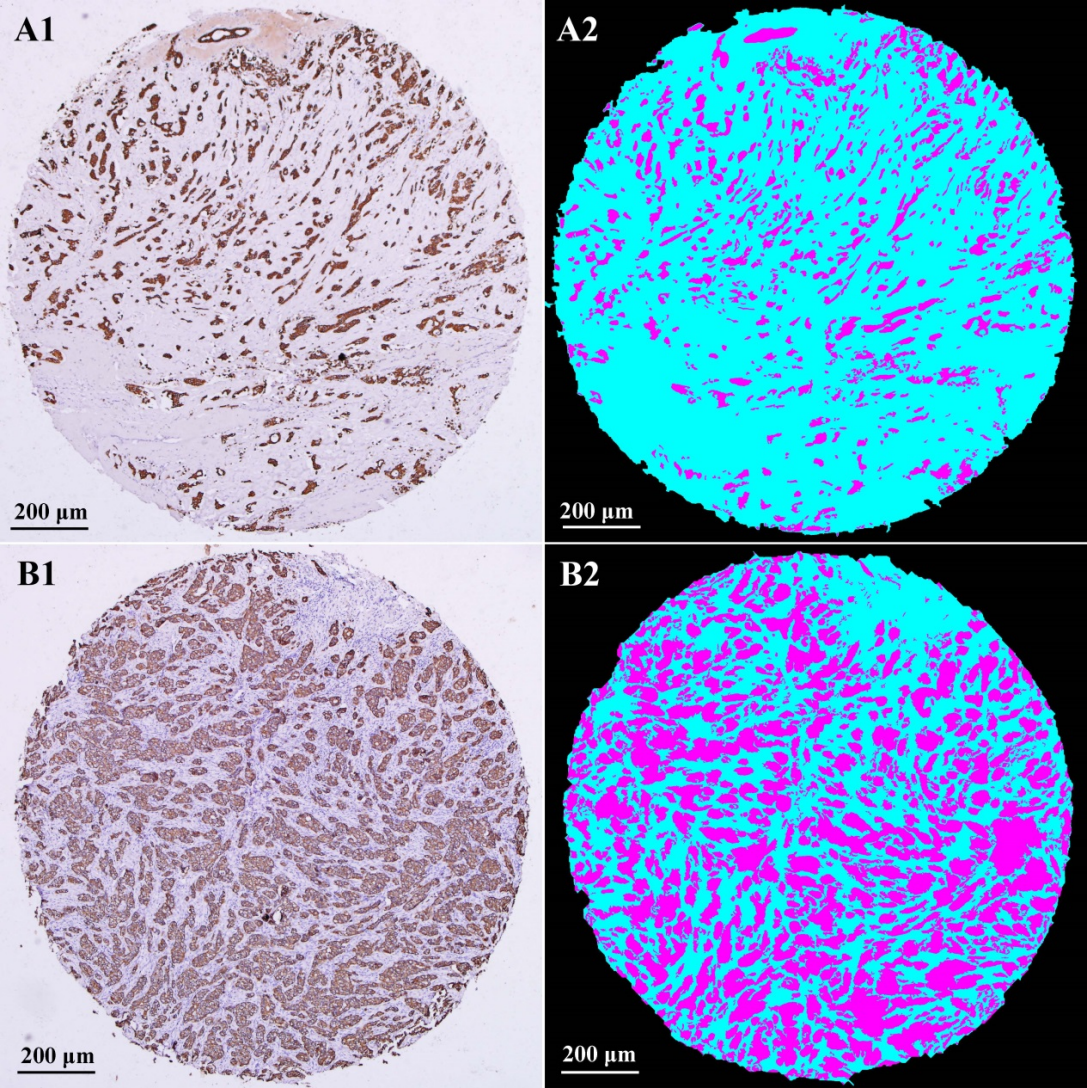
IHC staining images and computerized assessment results in TMAs. IHC staining of CK could specifically label tumor areas with clear contrast (**A1** and **B1**). After computerized recognition, tumor areas were marked in magenta, stromal areas in cyan, and none-cell areas in black (**A2** and **B2**). Examples of high stroma (**A1** and **A2**); Examples of low stroma (**B1** and **B2**). IHC: immunohistochemistry; CK: cytokeratin; TSR: tumor-stromal ratio.

**Figure 3:**
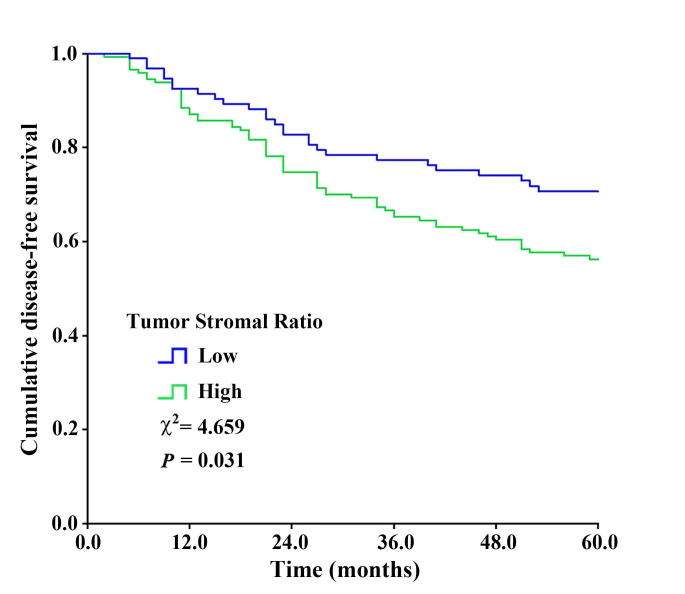
The Kaplan-Meier survival curve showing disease-free survival after stratification by TSR. High stroma was associated with worse 5-year disease free survival (χ^2^=4.659, *P* = 0.031) compared with low stroma.

**Figure 4:**
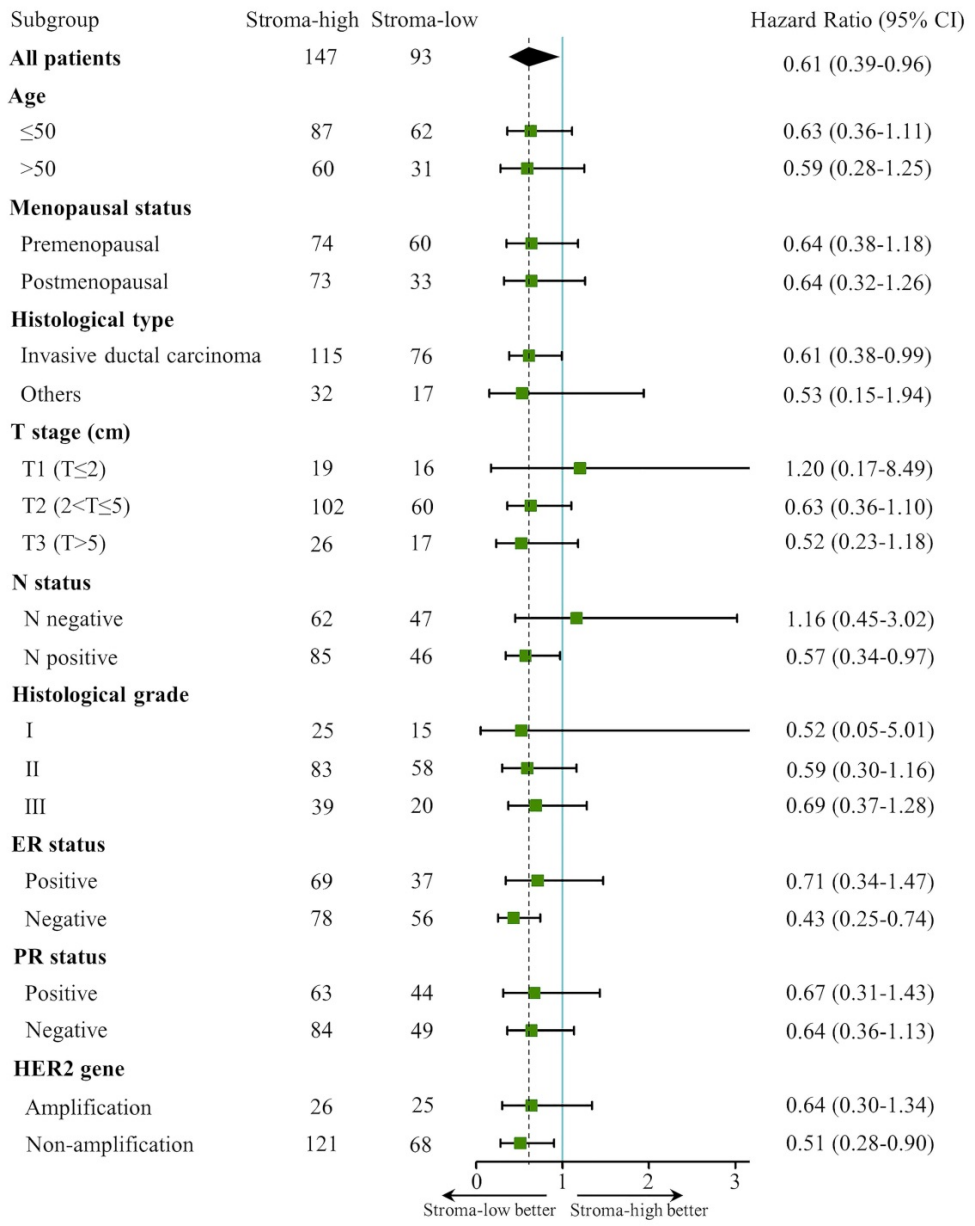
The forest plot of TSR associated with 5-DFS in subgroups. The dashed line represents the hazard ratio 0.61 in all patients. CI: confidence interval.

**Figure 5:**
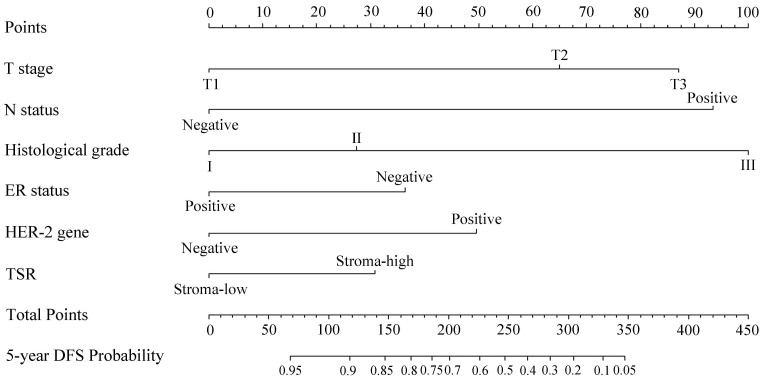
Nomogram predicting 5-year DFS probability of invasive BC patients.

**Figure 6:**
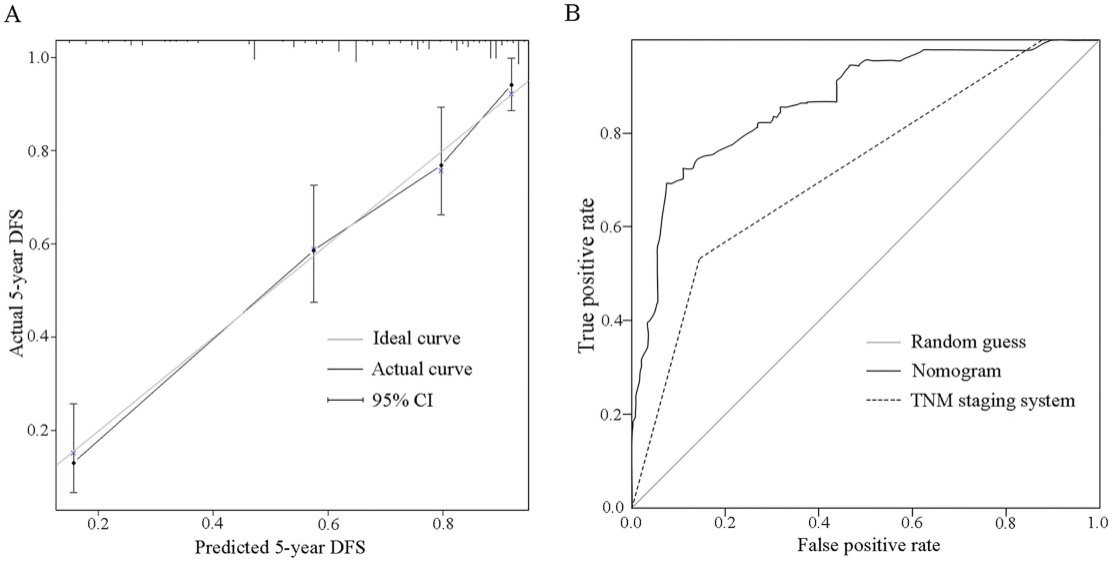
Calibration plot and ROC curve of the nomogram. **A**. On the calibration plot, the grey line represents an ideal evaluation, whereas the black line represents the performance of the nomogram, which showed favorable agreement between the predicted rate and actual rate. **B**. On the ROC curve, AUC of the nomogram (0.870 (95% CI: 0.823-0.917)) is greater than the TNM staging system (0.723 (95% CI: 0.657-0.790)).

**Figure 7:**
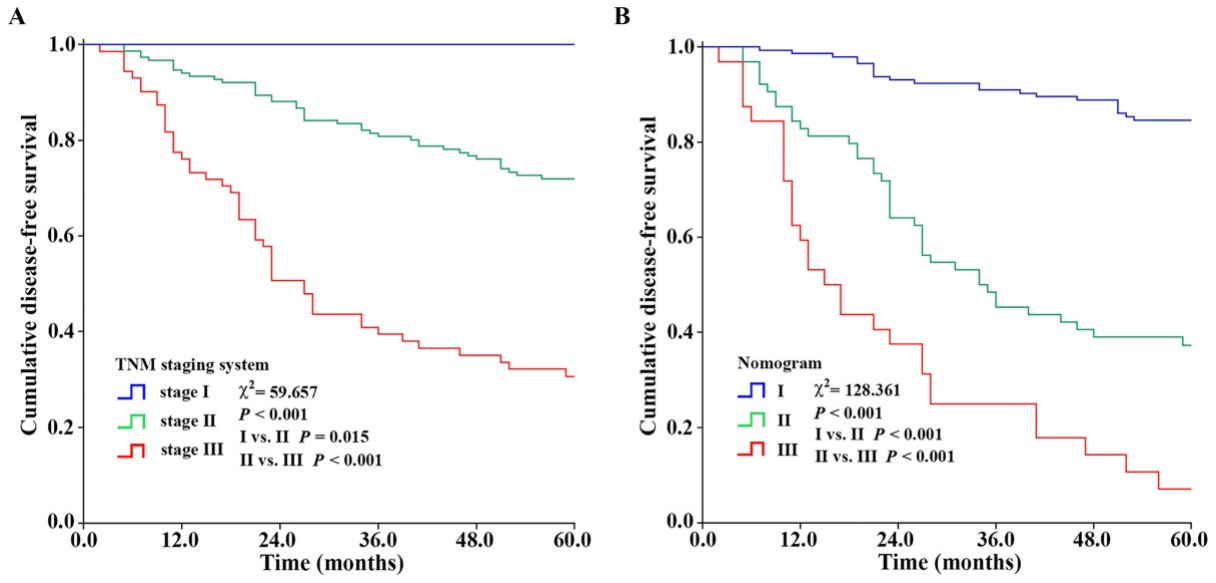
Kaplan-Meier survival curves showing the stratification of the nomogram and TNM staging system. The nomogram had a better ability to distinguish BC patients into three groups with different prognoses (χ^2^=128.361, *P* < 0.001 for I/II and II/III) than the TNM staging system (χ^2^=59.657, *P* = 0.015 for I/II, *P* < 0.001 for II/III).

**Figure 8:**
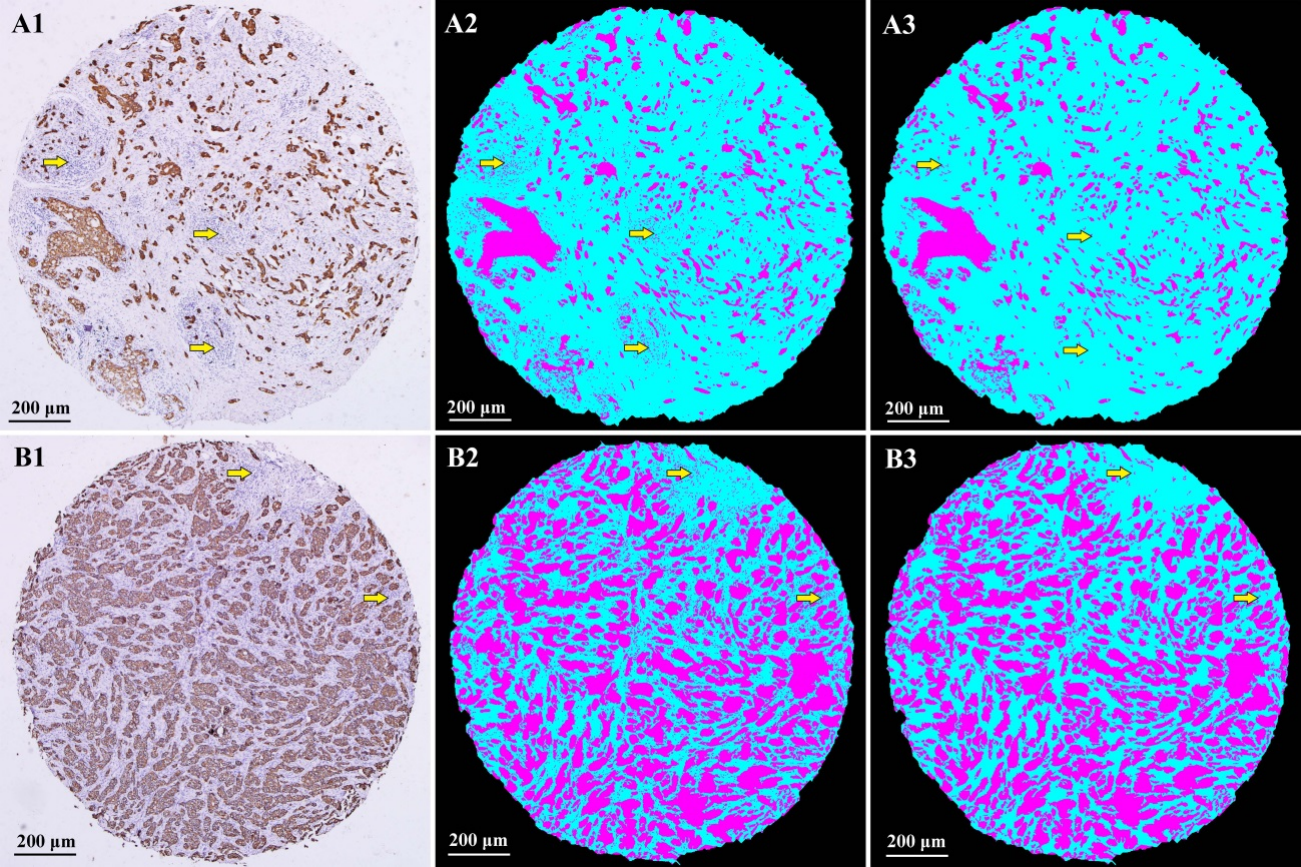
Comparison of recognition results by different thresholds. **Panels A2, B2**, a lower threshold was set, part of the cell nuclei of stroma cells stained in blue by hematoxylin were mistakenly identified as tumor objects. **Panels A3, B3**, a suitable threshold was set to eliminate small objects like the cell nuclei.

These two methods have been applied in various studies, which assessed TSR using HE staining slides and could be easily performed in routine pathology diagnostics 25-26. However, sometimes the boundary of tumor nests cannot be accurately identified due to low contrast between the tumor and stroma in HE staining, which makes it difficult for observers to perform the TSR assessment. In our previous study, IHC staining of CK was utilized to make a clear color contrast of brown tumor areas and off-white stromal areas, which makes it easier to assess TSR through visual scoring. However, with Cohen's kappa value of 0.77, the manual method was still criticized due to its intra- and inter-observer variations 11. To improve this disadvantage, we developed a computerized tumor/stroma recognition method. The computerized method with optimal reproducibility could enable the objective and standardized TSR quantification. The procedures were briefly demonstrated in **Fig. 1C**. Two major steps were performed to recognize the tumor object and the whole core object, respectively. In the final image, tumor areas were marked in magenta, stromal areas in cyan, and none-cell areas in black. TSR was automatically calculated as the area of stroma divided by the area of the whole core (tumor and stroma). Compared with similar studies of computer aided-recognition methods 27-29, several technical improvements have been made in our study. Firstly, as mentioned above, the key for computerized TSR assessment is to identify the boundary of tumor nests more accurately. To maximally differentiate any obscure boundaries, edge and sobel operators were utilized to make the calculated gradient value more accurate, so as to get clear contours of tumor objects. Secondly, the cell nuclei of stroma cells are stained in blue by hematoxylin, which may be mistakenly identified as tumor objects. To prevent this, in the morphological operation (dilate-> fill-> erode), a suitable threshold was set to eliminate small objects like the cell nuclei. Comparison of recognition results by different thresholds are shown in **Fig. 8**. Thirdly, combing with a high throughput approach, massive digital images of cores in TMAs could be assessed to calculate the proportion of stromal areas. It eliminated the onerous time and workflow required by visual scoring performed by experienced pathologists. In addition, this computerized tumor/stroma recognition method can also be applied to TSR assessment on microscopic fields under 10x objective or 20x objective, which is shown in **Fig. S2**. In routine pathology diagnostics, evaluation of the TSR started with microscopical orientation at low magnification. Subsequently, a high magnification was used in the selected areas. Through computerized assessment, the TSR can be accurately determined, rather than scored per tenfold increments.

So far, various studies have reported the prognostic value of TSR in different types of BC, for example, in triple-negative BC 30-31, lymph node-negative BC 32, primary operable invasive ductal BC 33, estrogen receptor-positive BC 24, and inflammatory BC 26. Most of them have shown an association between high stroma content and a poor prognosis. This study aimed at exploring the prognostic value of the TSR in invasive BC using CK-staining TMAs and computerized assessment. Consistent with previous studies from central Chinese 34-35, there was a high proportion of patients with lymph node-positivity and hormone receptor-negativity, which indicated more patients with aggressive BC and less patients qualified for endocrine therapy. As a result, prognosis of the subjects is poorer than that of common patients with invasive breast cancer. Among 240 specimens, 38.8% were determined as stroma-low and 61.2% as stroma-high through computerized assessment, and a worse DFS was found for patients with high stroma as compared to patients with low stroma (*χ2*=4.659, *P* = 0.031), with 5-year disease free survival rate of, respectively, 56.5 vs. 71.0%. Subgroup analysis demonstrated that the TSR was significantly associated with 5-DFS in invasive ductal carcinoma, N positive, ER negative, and HER-2 gene non-amplification groups.

The gold standard for prognostication in oncology remains the TNM staging system. It creates a system with a finite number of stages. For an individual patient, a higher TNM stage corresponds to a worse prognosis, but a concrete incidence rate or survival rate cannot be immediately determined. In addition, patients with the same anatomical spread yet variable outcomes (recurrence or survival) are categorized into the same stage, and have the same prognosis, which results in heterogeneity 36-37. Correspondingly, the nomogram, as a statistical predictive model, has emerged as a simpler, yet more advanced method 38-39. It assigns relative risk score to each predictor according to its contribution for the prognosis, and predicts the incidence rate or survival rate through a scoring system. Through the univariate and multivariate Cox regression analysis, variables including T stage, N status, histological grade, ER status, HER2 gene and TSR were selected into the best-fit model. The clinical availability of these six variables were examined, and then a nomogram was constructed to predict the probability of disease-free survival at the follow-up of 5 years for invasive BC patients. The nomogram showed favorable predictive performance, which included the discrimination ability quantified by the C-index (0.817, 95% CI: 0.775-0.858) and the calibration ability evaluated by the calibration plot. In addition, the results of C-index and AUC demonstrated that the predictive performance of the nomogram was superior to included independent prognostic factors and the TNM staging system.

The ability to categorize patients into different risk groups accurately is equally important because this is the premise of formulating treatment strategies. As a result, the sum of the covariate points was calculated for each patient, and a risk stratification based on the nomogram was established. Compared with the TNM staging system (χ^2^ = 59.657, *P* = 0.015 for I/II, *P* < 0.001 for II/III), the nomogram had a better ability to distinguish BC patients into three groups with different prognoses (χ^2^=128.361, *P* < 0.001 for I/II and II/III). Given these statistical results, we believed that this nomogram had adequate power of discrimination, calibration, and satisfactory risk stratification.

However, there are still several limitations in our study. Firstly, it is a retrospective research with a relatively small sample capacity, which may compromise the predictive performance of the nomogram. It will be valuable to conduct a prospective study with a larger sample. Secondly, although TMAs were constructed using standard procedures that only the most invasive tumor areas containing both tumor cells and tumor stroma were identified, not every core of TMAs can completely represent the optimal site for TSR assessment. More tumor cores taken from each specimen may reduce the selection bias. Thirdly, available external data for external validation of the nomogram is lacked. A supplementary of external data can help evaluate the external applicability of the nomogram.

## Conclusions

In general, our study established a computerized method to automatically assess the TSR in invasive BC using CK stained TMAs, and demonstrates that invasive BC patients of low TSR have poor prognoses. Furthermore, a nomogram containing TSR for predicting survival in invasive BC was established and assessed, which provided a comprehensive individualized risk prediction strategy, and might assist clinicians to make optimal care decisions for BC patients.

## Supplementary Material

Supplementary figures and tables.

## Figures and Tables

**Table 1:** The relationship between TSR and major clinico-pathological characteristics.

Characteristics	Total, n (%)	Stroma-high, n (%)	Stroma-low, n (%)	*P* value
**Age (years)**				0.244
≤ 50	149 (62.1)	87 (59.2)	62 (66.7)	
> 50	91 (37.9)	60 (40.8)	31 (33.3)	
**Menopausal status**				0.031
Premenopausal	134 (55.8)	74 (50.3)	60 (64.5)	
Postmenopausal	106 (44.2)	73 (49.7)	33 (35.5)	
**Histological type**				
Invasive ductal carcinoma	191 (79.6)	115 (78.2)	76 (81.7)	0.514
Others	49 (20.4)	32(21.8)	17(18.3)	
**T stage (cm)**				0.629
T1 (T ≤ 2)	35 (15.0)	19 (12.9)	16 (17.2)	
T2 (2 < T ≤ 5)	162 (67.5)	102 (69.4)	60 (64.5)	
T3 (T > 5)	43 (17.5)	26 (17.7)	17 (18.3)	
**N status**				0.205
N negative	109 (45.4)	62 (42.2)	47 (50.5)	
N positive	131 (54.6)	85 (57.8)	46 (49.5)	
**Histological grade**				0.622
I	40 (16.7)	25 (17.0)	15 (16.1)	
II	141 (58.8)	83 (56.5)	58 (62.4)	
III	59 (24.6)	39 (26.5)	20 (21.5)	
**ER status^a^**				0.277
Positive	106 (44.2)	69 (46.9)	37 (39.8)	
Negative	134 (55.8)	78 (53.1)	56 (60.2)	
**PR status^a^**				0.499
Positive	107 (44.6)	63 (42.9)	44 (47.3)	
Negative	133 (55.4)	84 (57.1)	49 (52.7)	
**HER2 gene^b^**				
Amplification	51 (21.3)	26 (17.7)	25 (26.9)	0.090
Non-amplification	189 (78.7)	121 (82.3)	68 (73.1)	

^a^ER, PR was determined by immunohistochemistry staining according to guideline 19; ^b^HER2 gene was determined by fluorescent *in-situ* hybridization (FISH) according to guideline 20. BC: breast cancer; T: tumor; N: node; TSR: tumor-stromal ratio; ER: estrogen receptor; HER2: human epidermal growth factor receptor-2.

**Table 2:** Multivariable analysis for 5-DFS and the C-index for single predictors.

Parameters	Multivariable analysis			C-index (95% CI)
	HR	95%CI	P valuea	
**T stage (cm)**				0.625 (0.576-0.675)
T1 (T≤2)	1.000			
T2 (2<T≤5)	2.576	0.925-7.171	0.070	
T3 (T>5)	3.506	1.199-10.251	0.022	
**N status**				0.678 (0.635-0.721)
Negative	1.000			
Positive	3.854	2.201-6.747	< 0.001	
**Histological grade**				0.684 (0.639-0.729)
I	1.000			
II	1.479	0.513-4.262	0.469	
III	4.250	1.413-12.782	0.010	
**ER status**				0.611 (0.563-0.659)
Negative	1.000			
Positive	0.568	0.325-0.995	0.048	
**PR status**				
Negative	1.000			
Positive	1.076	0.640-1.807	0.783	
**HER2 gene**				0.588 (0.541-0.635)
Non-amplification	1.000			
Amplification	2.045	1.258-3.325	0.004	
**TSR**				0.555 (0.505-0.605)
Stroma-high	1.000			
Stroma-low	0.643	0.399-1.035	0.069	

T: tumor; N: node; TSR: tumor-stromal ratio; ER: estrogen receptor; PR: progesterone receptor; HER2: human epidermal growth factor receptor-2.
