# Supplementary material for: Computerized Assessment of the Tumor-stromal Ratio and Proposal of a Novel Nomogram for Predicting Survival in Invasive Breast Cancer
Source: J Cancer. 2021 Apr 19;12(12):3427–38. doi: 10.7150/jca.55750 (PMC8120167; doi:10.7150/jca.55750)
Supplement: Supplementary file 1 — Supplementary figures and tables. [file jcav12p3427s1.pdf]

Supplementary Table 1 Analysis of characteristics regarding 5-DFS

Characteristics	Total (N=240)	No. of disease (%)	5-DFS rate (%)	P value
**Age (years)**				0.182
≤ 50	149 (62.1)	55 (60.4)	63.1	
> 50	91 (37.9)	36 (39.6)	60.4	
**Menopausal status**				<0.001
Premenopausal	134 (55.8)	45 (49.5)	66.4	
Postmenopausal	106 (44.2)	46 (50.5)	56.6	
**Histological type**				<0.001
Invasive ductal carcinoma	191 (79.6)	78 (85.7)	59.2	
Others	49 (20.4)	13 (14.3)	73.5	
**T stage (cm)**				<0.001
T1 (T ≤ 2)	35 (15.0)	4 (4.4)	88.6	
T2 (2 < T ≤ 5)	162 (67.5)	60 (65.9)	63.0	
T3 (T > 5)	43 (17.5)	27 (28.7)	37.2	
**N status**				<0.001
N negative	109 (45.4)	17 (18.7)	84.4	
N positive	131 (54.6)	74 (81.3)	43.5	
**Histological grade**				<0.001
I	40 (16.7)	4 (4.4)	90.0	
II	141 (58.8)	40 (43.9)	71.6	
III	59 (24.6)	47 (51.7)	20.3	
**ER status**				<0.001
Positive	106 (44.2)	25 (27.5)	76.4	
Negative	134 (55.8)	66 (72.5)	50.7	
**PR status**				<0.001
Positive	107 (44.6)	31 (33.7)	71.0	

Negative	133 (55.4)	61 (66.3)	54.1	
**HER2 gene**				<0.001
Amplification	51 (21.3)	29 (31.9)	43.1	
Non-amplification	189 (78.7)	62 (68.1)	67.2	

T: tumor; N: node; 5-DFS: 5-year disease free survival; ER: estrogen receptor; PR: progesterone receptor; HER2: human epidermal growth factor receptor-2

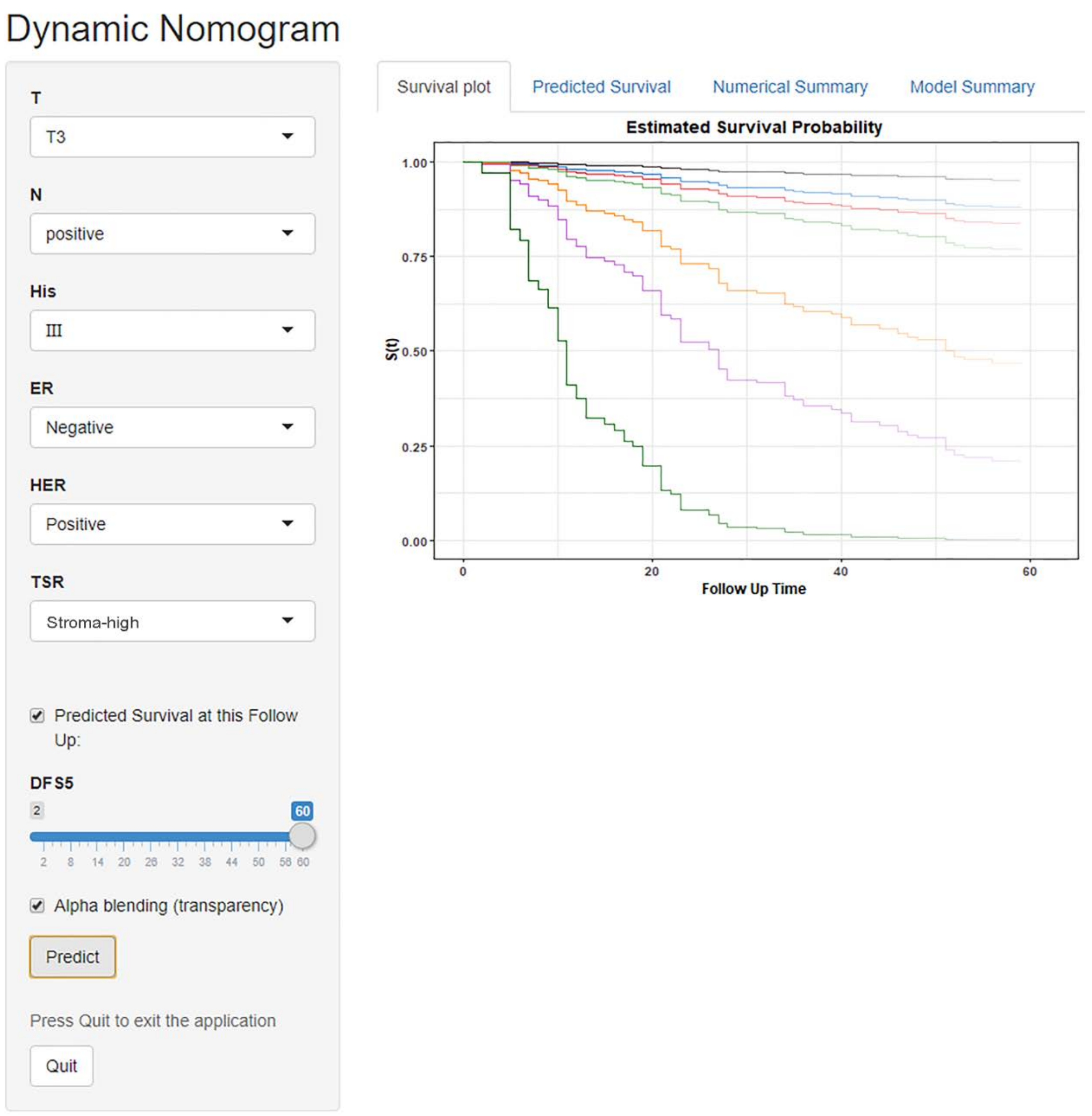

Fig. S1 A dynamic version of the Nomogram

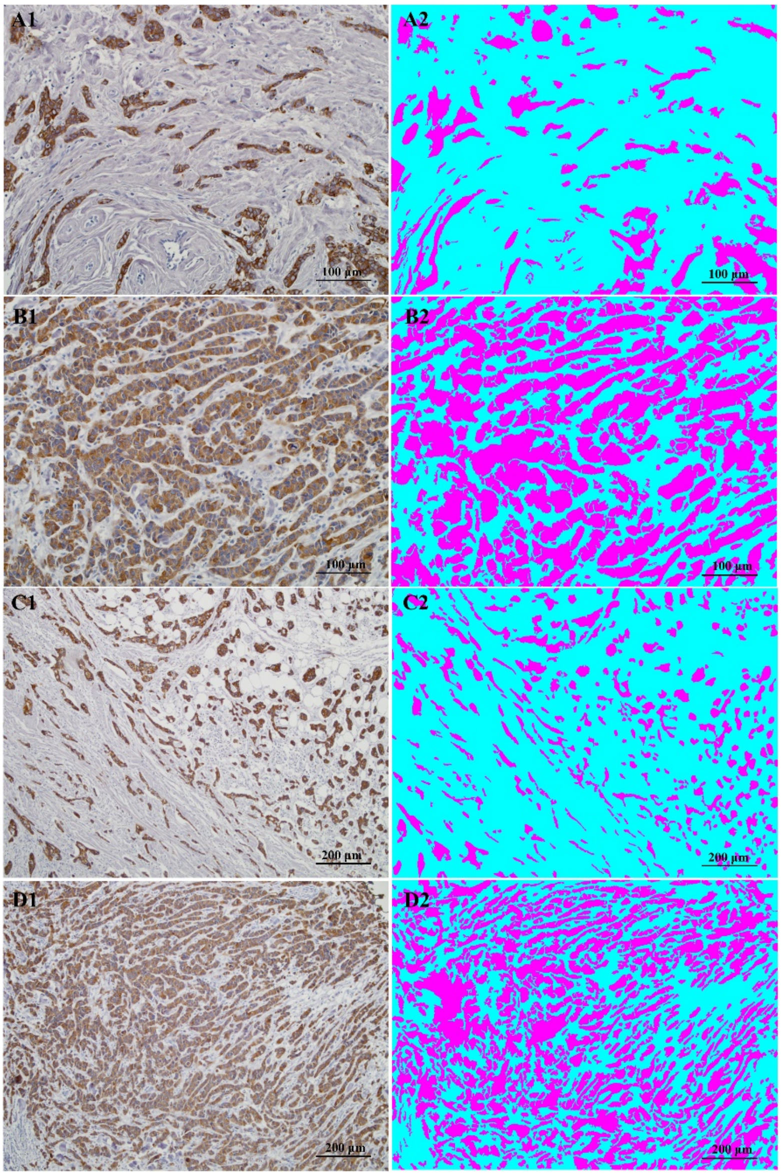

Fig. S2 IHC staining images and computerized assessment results on microscopic fields. **Panels A1, A2** are examples of high stroma, with an estimated TSR of 83.9% (20× objective, scale bar=100μm). **Panels B1, B2** are examples of low stroma, with an estimated TSR of 52.6% (20× objective, scale bar=100μm). **Panels C1, C2** are examples of high stroma, with an estimated TSR of 83.4% (10× objective, scale bar=200μm). **Panels D1, D2** are examples of low stroma, with an estimated TSR of 45.4% (10× objective, scale bar=200μm).
